# Human Cytomegalovirus Host Interactions: EGFR and Host Cell Signaling Is a Point of Convergence Between Viral Infection and Functional Changes in Infected Cells

**DOI:** 10.3389/fmicb.2021.660901

**Published:** 2021-05-07

**Authors:** Byeong-Jae Lee, Chan-Ki Min, Meaghan Hancock, Daniel N. Streblow, Patrizia Caposio, Felicia D. Goodrum, Andrew D. Yurochko

**Affiliations:** ^1^Department of Microbiology & Immunology, Center for Molecular and Tumor Virology, Louisiana State University Health Sciences Center Shreveport, Shreveport, LA, United States; ^2^Center for Applied Immunology and Pathological Processes, Louisiana State University Health Sciences Center Shreveport, Shreveport, LA, United States; ^3^Center of Excellence for Emerging Viral Threats, Louisiana State University Health Sciences Center Shreveport, Shreveport, LA, United States; ^4^Vaccine and Gene Therapy Institute, Oregon Health & Science University, Beaverton, OR, United States; ^5^BIO5 Institute, University of Arizona, Tucson, AZ, United States; ^6^Feist-Weiller Cancer Center, Louisiana State University Health Sciences Center Shreveport, Shreveport, LA, United States; ^7^Center for Cardiovascular Diseases and Sciences, Louisiana State University Health Sciences Center Shreveport, Shreveport, LA, United States; ^8^Center of Excellence in Arthritis and Rheumatology, Louisiana State University Health Sciences Center Shreveport, Shreveport, LA, United States

**Keywords:** human cytomegalovirus (HCMV), monocytes, progenitor cells, epidermal growth factor receptor (EGFR), glycoproteins, cell signaling, differentiation, latency

## Abstract

Viruses have evolved diverse strategies to manipulate cellular signaling pathways in order to promote infection and/or persistence. Human cytomegalovirus (HCMV) possesses a number of unique properties that allow the virus to alter cellular events required for infection of a diverse array of host cell types and long-term persistence. Of specific importance is infection of bone marrow derived and myeloid lineage cells, such as peripheral blood monocytes and CD34^+^ hematopoietic progenitor cells (HPCs) because of their essential role in dissemination of the virus and for the establishment of latency. Viral induced signaling through the Epidermal Growth Factor Receptor (EGFR) and other receptors such as integrins are key control points for viral-induced cellular changes and productive and latent infection in host organ systems. This review will explore the current understanding of HCMV strategies utilized to hijack cellular signaling pathways, such as EGFR, to promote the wide-spread dissemination and the classic life-long herpesvirus persistence.

## Introduction

Human cytomegalovirus (HCMV) infection is a significant public health threat worldwide (Stagno et al., 1986; Bentz et al., 2006; Arvin, 2007; Nogalski et al., 2014; Fulkerson et al., 2021). HCMV is a leading cause of morbidity and mortality in developing fetuses and the immunocompromised population (Stagno et al., 1986; Bentz et al., 2006; Arvin, 2007; Nogalski et al., 2014; Fulkerson et al., 2021). HCMV infection in neonates is the leading cause of congenital central nervous system damage and deafness (Boppana et al., 2011). HCMV is also a leading infectious agent negatively affecting the outcome of solid organ and bone marrow transplants. In healthy individuals, HCMV infection can cause mononucleosis and is associated with some vascular diseases and cancers (Soderberg-Naucler, 2008; Pass et al., 2009; Wang et al., 2017; Styczynski, 2018; Gerna and Lilleri, 2020).

A hallmark of HCMV infection is wide-spread dissemination of the virus from the blood in monocytes to most organs and then the establishment of latency in the bone marrow in progenitor cells that in turn, during reactivation, differentiate into monocytes to again disseminate the virus to host organ tissue (Sinzger et al., 1995; Smith et al., 2004a; Stevenson et al., 2014; Collins-McMillen et al., 2018b). Therefore, one of the first essential steps for hematogenous dissemination is the infection of monocytes (Smith et al., 2004b; Yurochko, 2008; Chan et al., 2010, 2012a; Nogalski et al., 2011). The virus, in turn, hijacks infected monocyte signaling pathways in order to promote efficient viral spread to most peripheral organs and the bone marrow (Smith et al., 2004b; Yurochko, 2008; Chan et al., 2010, 2012a; Nogalski et al., 2011). Furthermore, infiltration of infected monocytes into host organ systems allows for viral spread to additional hosts through release of new virus in a variety of body fluids (such as in saliva and urine) and spread to the bone marrow where latency is established in CD34^+^ hematopoietic progenitor cells (HPCs). The CD34^+^ HPCs during reactivation differentiate along myeloid lineage development pathways to become monocytes allowing organ dissemination and additional host to host spread (Goodrum et al., 2002; Kim et al., 2017; Collins-McMillen et al., 2018a). This review examines the processes of HCMV infection of these two critical cells and specifically how viral signaling through cellular receptors is a key molecular trigger for HCMV infection and persistence.

Human cytomegalovirus must first attach to the cell via an interaction between viral membrane glycoproteins and specific cell surface receptor-binding molecules (Chan et al., 2009b, 2012a; Collins-McMillen et al., 2017a). Several viral glycoprotein complexes are crucial for viral entry/internalization of monocytes and other cell types, two of which (gB and the gH complexes) will be focused on extensively in this report. gB and the gH/gL complexes help dictate infection tropism and serve as central ligands for viral attachment to clinically relevant cell types (Nogalski et al., 2013). The gB and the gH/gL complexes engage the cell through the epidermal growth factor receptor (EGFR) and cellular integrins, respectively (Chan et al., 2009b; Nogalski et al., 2011, 2013; Kim et al., 2016). This receptor-ligand engagement in turn activates cellular signaling and the functional changes in the target monocytes to promote both productive infection and persistence in host organ systems.

To contact a target host cell, the viral ligands in the envelope, gB (UL55), gH (UL75)/gL (UL115)/gO (ULl74), which make up the gH/gL/gO complex or the trimer, and the gH/gL/UL128-131 complex or the pentamer, and gM (UL100)/gN (UL73) interact with cellular receptors that in turn lead to the activation of various signal molecules in the infected host cells (Mach et al., 2005; Shimamura et al., 2006; Shen et al., 2007; Fields et al., 2013; Stegmann et al., 2017a; Liu et al., 2018; Nguyen et al., 2018; Gerna et al., 2019; Nishimura and Mori, 2019). There are some receptors that seem to be present on most infected cell types and these include heparan sulfate proteoglycans, cellular integrins (α2β1, α6β1, and αvβ3) (Feire et al., 2004; Wang et al., 2005) and Toll-like receptors (Compton et al., 2003; Boehme et al., 2006), while other receptors such as EGFR (Wang et al., 2003, 2005), are only found on some cell types and may help dictate tropism. Other potential tropism receptors include the Platelet-Derived Growth Factor Receptor [PDGFR (Wu et al., 2017; Nguyen and Kamil, 2018; Wu et al., 2018; Nishimura and Mori, 2019)], Neuropilin-2 [Nrp-2 (Martinez-Martin et al., 2018)], and Olfactory Receptor Family 14 Subfamily I Member 1 [OR14I1 (Xiaofei et al., 2019)]. In general, whether these receptors are specific for a specific cell type or are more conserved across a variety of cell types, they are important for viral attachment, internalization, and fusion and entry into the cytoplasm of the infected cell (Nowlin et al., 1991; Navarro et al., 1993; Boyle and Compton, 1998; Compton, 2000; Isaacson and Compton, 2009). They also all seem to initiate multiple downstream cellular signaling pathways during infection (Nogalski et al., 2007; Smith et al., 2007; Yurochko, 2008; Reeves et al., 2012).

As an extended introduction, we would like to emphasize why we chose to focus primarily on monocytes and CD34^+^ HPCs in this review. Both cell types are important targets for HCMV infection and are critical to life-long persistence and dissemination within an individual infected host and the general human population. Monocytes are essential for hematogenous dissemination of HCMV to multiple organ systems following primary infection (Nogalski et al., 2011). That is, HCMV uses the biological features of monocytes: motility, ability to migrate to all tissue types and differentiation into long-lived macrophages (Nogalski et al., 2012; Stevenson et al., 2014; Collins-McMillen et al., 2017b, 2018b) to promote initial dissemination to the multiple host organs that serve as the source of viral spread from host to host and for the eventual establishment of the viral persistence/latency seen in the bone marrow in CD34^+^ HPCs. Monocytes also seem to play an essential role in the establishment of viral latency through this initial spread and then in the organ dissemination following reactivation in CD34^+^ HPCs and the differentiation of these CD34^+^ HPCs into monocytes (Stevenson et al., 2014; Collins-McMillen et al., 2017b, 2018a; Kim et al., 2017). Like infection of monocytes, initial infection of CD34^+^ HPCs relies on receptor-ligand engagement with EGFR engagement being key to early infection (Kim et al., 2016). Although we did not focus on the receptor-ligand engagement for different cell types, distinct downstream signaling pathways, and functional consequences in this review, it also occurs during the infection of epithelial, endothelial cells, fibroblasts, and others (Sinzger et al., 1995; Chan et al., 2008b; Yurochko, 2008).

For HCMV to initially infect monocytes, the virus must deal with several biological barriers, and one such barrier is the short lifespan of blood monocytes and the initial lack of viral gene expression and replication in these cells. HCMV-infected monocytes are initially short lived and are non-permissive for productive infection Yurochko et al., 1989, 1990, 1992, 1995, 1997a,b, 1999; Taylor-Wiedeman et al., 1991; Mendelson et al., 1996; Hahn et al., 1998; Yurochko and Huang, 2000; Chan et al., 2010). To regulate these processes, HCMV utilizes a unique signalosome generated initially by the signaling downstream of gB/EGFR and pentamer/β1 and β3 integrin engagement at the cell surface and then activation of EGFR and integrin dependent events post entry (Collins-McMillen et al., 2017b; Fulkerson et al., 2020). This signalosome, as discussed in more detail below, alters key biological steps in infected monocyte motility (Smith et al., 2004b; Chan et al., 2009a) and survival through manipulation of Bcl-2 family members (Chan et al., 2010; Collins-McMillen et al., 2016). In addition, this viral ligand-induced signalosome promotes monocyte-to-macrophage differentiation and polarization of these differentiated macrophages (Yurochko et al., 1989; Stevenson et al., 2014; Collins-McMillen et al., 2017a, b, 2018b). Because macrophages are naturally long-lived cells and once differentiated can produce and shed virus for weeks to months (Smith et al., 2004b; Stevenson et al., 2014), they can serve as a source of organ persistence for the virus and shedding in bodily fluids to other hosts. Although distinct, latent infection of CD34^+^ HPCs also produces a unique signalosome that is essential for the establishment of, maintenance of, and reactivation from latency and the associated differentiation of these progenitor cells into monocytes (Goodrum et al., 2002; Streblow and Nelson, 2003). We hope to shed light on HCMV signaling through key cellular receptors, and how that signaling initiates productive infection and persistence in this review.

## HCMV Receptors and Ligand-Induced Signaling Pathway and Cells

### HCMV Glycoproteins

**FIGURE 1:**
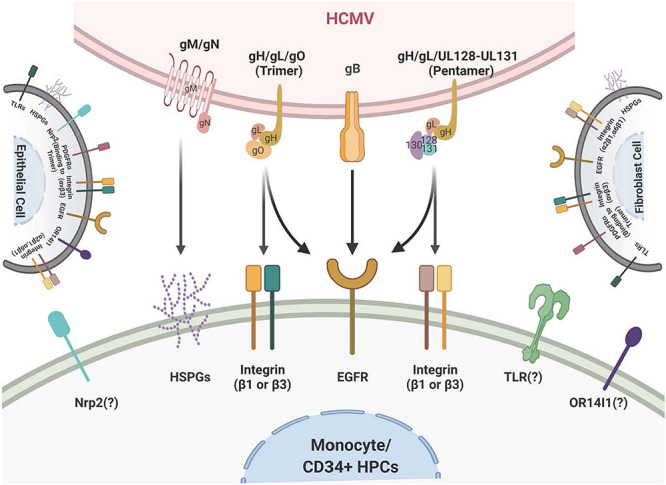
Schematic of the HCMV glycoprotein complexes and their cognate receptors. HCMV attachment and entry into monocytes depends on several envelope glycoprotein complexes including gM/gN, gB, and the gH complexes. gM/gN mediates initial attachment via glycosaminoglycans. gB is known to interact with the epidermal growth factor receptor (EGFR) on monocytes. gB may also interact with integrins on monocytes, although this is unresolved. The trimeric gH/gL/gO complex interacts with integrins and the platelet-derived growth factor receptor alpha (PDGFRα) on fibroblasts; at present the trimer only has been reported to interact with integrins on monocytes. The pentameric gH/gL/UL128-131 complex interacts with β1- and β3-integrins on monocytes. Several other receptors, such as OR14I1 and Nrp2, have been reported to bind to the pentamer; this engagement remains unresolved during infection of monocytes. The membrane of fibroblasts is illustrated on the right and the membrane of epithelial cells is illustrated on the left and they include the multiple receptors that have been reported to engage the HCMV glycoprotein complexes during infection of fibroblasts and epithelial cells, respectively.

Human cytomegalovirus is a species-specific β-herpesvirus that infects an extensive range of cell types, including monocytes, macrophages, endothelial cells, epithelial cells, vascular smooth muscle cells, stromal cells, neuronal cells, fibroblasts, CD34^+^ HPCs, and hepatocytes (Sinzger et al., 1995; Jarvis and Nelson, 2002). Although HCMV can infect many different cell and tissue types, the virus has a set number of viral entry complexes that can be used, which means the virus has adapted to utilize these complexes in a way that promotes efficient transmission and infection of different cell types in the new host to allow for lifelong persistence (see Figure 1 for a cartoon of viral glycoproteins and their cognate ligands on monocytes/CD34^+^ HPCs and other cell types). HCMV gB, like all herpesvirus gBs, is essential for viral membrane fusion with cellular surface or vesicular membranes (Compton et al., 1992; Navarro et al., 1993; Tugizov et al., 1994; Vanarsdall et al., 2008; Isaacson and Compton, 2009; Zhou et al., 2015) and in an HCMV-specific manner, HCMV gB is a receptor-ligand binding protein for EGFR (Wang et al., 2003, 2005; Chan et al., 2012a; Fulkerson et al., 2020). HCMV gB is synthesized as a 160 kDa precursor that is cleaved by furin in the Golgi, resulting in 116 and 55 kDa fragments that become disulfide-linked to make the mature gB (Britt and Auger, 1986). This mature gB is what engages several cell-surface proteins, including the primary receptor, EGFR (Wang et al., 2003; Chan et al., 2012a; Fulkerson et al., 2020), as well as perhaps PDGFR-α (Soroceanu et al., 2008) and some integrins (Bentz and Yurochko, 2008; Feire et al., 2010). In monocytes, this receptor likely helps determine tropism for blood leukocytes, as B cells and T cells do not express EGFR, correlating with the lack of infection of these cells (Chan and Yurochko, 2014). In addition, PDGFR is not reported to be expressed on freshly-isolated blood monocytes (Chan et al., 2009b) or freshly-isolated CD34^+^ HPCs (Su et al., 2002) suggesting that the gB/EGFR interaction plays a key role in the infection of these cell types. For this review and for infection of monocytes and CD34^+^ HPCs, gB engagement of EGFR is essential for entry and the post entry events related to nuclear translocation as well as for the associated downstream signaling (Chan et al., 2009b; Kim et al., 2017; Fulkerson et al., 2020).

There are multiple gH complexes, from the newly discovered gH interaction with UL116 [gH (UL75)/UL116] (Caló et al., 2016) to the known gH complexes of gH/gL (UL115)/gO (UL174) or the trimer (Stegmann et al., 2017a) and gH/gL/UL128-131 or the pentamer (Nguyen and Kamil, 2018; Nishimura and Mori, 2019). These unique complexes directly affect cell-type-specific entry. For example, fibroblast entry processes are largely mediated by the glycoproteins/glycoprotein complexes gB, gH/gL/gO (the trimer), and gM (UL100)/gN (UL73), whereas entry into epithelial cells, endothelial cells, and monocytes/macrophages also require the gH/gL/UL128-131 pentameric complex (Hahn et al., 1998, 2004; Jarvis and Nelson, 2002; Ricciotti and Fitzgerald, 2011; Aoki and Narumiya, 2012; Stegmann et al., 2017b). The HCMV gM/gN complex (Mach et al., 2005) is the most abundant glycoprotein complex found on virions (Soroceanu et al., 2008; Feire et al., 2010) and has been shown to interact with heparin sulfate proteoglycans on the cell surface (Kari and Gehrz, 1992), and may also have some intracellular roles during viral replication (Pignatelli et al., 2003; Mach et al., 2005). HCMV UL116 has recently been shown to be required for the production of infectious virus and may help chaperone gH complexes into virions (Caló et al., 2016; Nguyen and Kamil, 2018; Gatault et al., 2021).

There are both shared and unique features of the gH/gL glycoprotein complexes among the different herpesviruses. For example, gH/gL (along with gB) comprise the central herpesvirus membrane fusion machinery (Weed and Nicola, 2017). However, other aspects of these complexes are unique to HCMV and/or the other β-herpesviruses. gO (and homologs) are found only in β-herpesviruses, and the gO-associated trimer is required for cellular infectivity of cell-free HCMV virions (Wille et al., 2010; Liu et al., 2018); however, its role in modulating signal transduction pathways in virus-infected host cells is not resolved. The pentameric complex is unique to HCMV (Wang and Shenk, 2005b; Ryckman et al., 2008; Ciferri et al., 2015; Chandramouli et al., 2017) and is required for infection of epithelial and endothelial cells, as well as leukocytes (Bentz et al., 2006; Kim et al., 2017). The complex is genetically unstable during HCMV passage in fibroblasts and many lab-adapted stains have lost this complex through mutation in one or more of the pentamer complex-specific glycoprotein coding sequences (Akter et al., 2003; Wille et al., 2010). Repairing the HCMV pentamer complex in a fibroblast-adapted laboratory strain restores infectivity of epithelial and endothelial cells, and monocytes (Wang and Shenk, 2005a, b; Nogalski et al., 2013). The pentamer binds to different cellular integrins and seems to have different integrin binding partners on different cell types (Nogalski et al., 2014; Collins-McMillen et al., 2017b; Vanarsdall et al., 2018). In addition, the pentamer was recently reported to bind to Nrp2 (Martinez-Martin et al., 2018) and OR14I1 (Xiaofei et al., 2019) on epithelial cells, perhaps providing epithelial cell tropism. The pentamer has also been documented to interact with thrombomodulin (Hsu et al., 2015; van den Boomen and Lehner, 2015). CD147 (Vanarsdall et al., 2018) and CD151 (tetraspanin) and THY-1 (CD90) have also been found to play a role during HCMV infection of some cell types (Hochdorfer et al., 2016). However, the biological relevance and importance of these receptors in infection monocytes or CD34^+^ HPCs and in cell signaling remain unresolved. Overall, viral glycoproteins are an indispensable cell type specific strategic weapon for successful viral infection via their receptor binding capabilities, their ability to induce cellular signaling in host cells and to allow viral entry into that target cell. Further studies are needed to determine the precise mechanisms by which the many HCMV glycoproteins and the many receptors together or independently alters host signaling pathways in the many cells that HCMV infects.

### EGFR and Integrins Are Essential Entry Receptors Required for Initiating Cellular Signal Pathway During HCMV Infection of Monocytes

#### Epidermal Growth Factor Receptor (EGFR) (See Figure 2)

**FIGURE 2:**
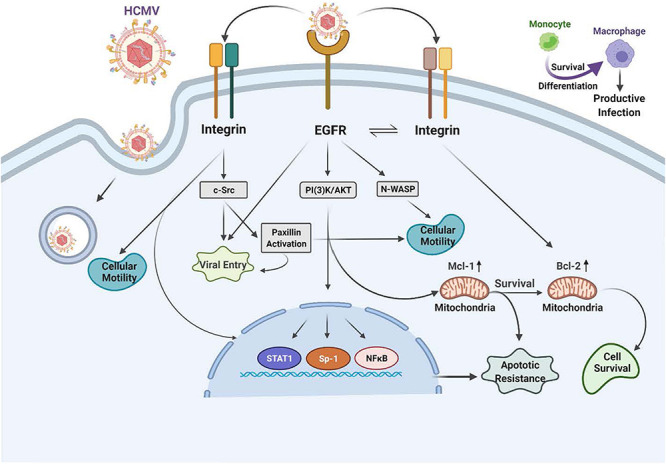
Human cytomegalovirus (HCMV) glycoprotein binding to cognate receptors controls viral signaling, trafficking and the key cellular functions required for productive infection of monocytes. The HCMV glycoproteins bind several cellular receptors, including EGFR and the β1- and β3-integrins. Following binding, these receptor engagements trigger a variety of signal transduction pathways in the infected monocyte. For example, gB binding to EGFR results in activation of the intrinsic EGFR tyrosine kinase and down stream signaling that is required for entry, for the unique multi-vesicular nuclear translocation process seen in monocytes, as well as for motility, and the survival of the monocytes and their differentiation into macrophages. The engagement of the pentameric complex, gH/gL/UL128-131 with β1- and β3-integrins on the surface of monocytes activates c-Src to drive the required signaling pathways needed for entry, nuclear translocation, motility, survival and differentiation. More specifically, this downstream signaling following EGFR and integrin engagement acts in concert to activate numerous down stream signaling pathways, including the NF-κB pathway, PI(3)K, and the MAPK pathway, which culminates in enhanced transactivation of many host cell promoters via increases in transcriptional regulators, altercations in actin regulatory proteins that modulate motility (such as N-WASP and paxillin) and changes in Mcl-1 and Bcl-2 to promote survival of short-lived monocytes.

The HCMV glycoproteins regulate the key intracellular signaling pathways in human primary monocytes required for productive infection and the establishment of persistence. Following the engagement of heparan sulfate proteoglycans, HCMV infection of monocytes begins via the interaction of gB and the gH/gL complexes with host cell surface EGFR and β1 and β3 integrins (Chan et al., 2009b, 2012a; Nogalski et al., 2011, 2013; Kim et al., 2016; Fulkerson et al., 2020). Epidermal growth factor receptor (EGFR or ErbB-1) is a member of the ErbB receptor tyrosine kinase family, which can bind epidermal growth factor (EGF) or transforming growth factor-alpha (TGF-α) (Wieduwilt and Moasser, 2008). Studies have revealed that downstream EGFR signaling can be unique and functionally distinct based on the ligand and the cell type expressing EGFR (Wieduwilt and Moasser, 2008). In support of this cell type specific EGFR biology during HCMV infection, one can note the differences between EGFR-dependent signaling seen in infected monocytes (Chan et al., 2009b, 2012a; Nogalski et al., 2011, 2013; Kim et al., 2016; Fulkerson et al., 2020) vs. that seen in infected endothelial cells (ECs) (Bentz and Yurochko, 2008) or infected trophoblasts (LaMarca et al., 2006). There are also reported differences in EGFR signaling in CD34^+^ HPCs (Kim et al., 2017) and that will be discussed in more detail below.

In monocytes, HCMV induces the phosphorylation of EGFR following viral binding and that engagement initiates the activation of PI(3)K and Akt. When considering other peripheral blood cell types, such as T cell and B cells, EGFR expression seems to be involved in the select myeloid tropism of the virus (Xiaofei et al., 2019). This HCMV-activated EGFR in monocytes promotes motility and transendothelial migration to allow virus-infected cells to gain entry into peripheral organ tissues (Smith et al., 2004a, b, 2007; Bentz et al., 2006; Chan et al., 2009b; Collins-McMillen et al., 2017a, b). Mechanistically, gB/EGFR engagement results in the upregulation and select usage of the Neural Wiskott–Aldrich Syndrome protein (N-WASP) as a mechanism to promote heightened motility (Chan et al., 2009b), as opposed to the use of the standard Wiskott–Aldrich Syndrome protein (WASP) that controls chemokine-induced motility in monocytes (Snapper and Rosen, 1999; Tsuboi, 2006; Chan et al., 2009b; Lee et al., 2017). That is, under normal circumstances, WASP usually helps modulate monocyte motility (Snapper and Rosen, 1999; Tsuboi, 2006; Chan et al., 2009b; Lee et al., 2017). WASP and N-WASP are actin nucleators that mechanistically drive changes in the actin cytoskeleton associated with cell movement. This use of N-WASP showcases some of the distinct properties of the gB/EGFR receptor-ligand engagement in monocytes. N-WASP is responsible for highly motile cancer cell movement (Yamaguchi and Condeelis, 2007; Kurisu and Takenawa, 2010; Frugtniet et al., 2015) and biochemically is more efficient at nucleating actin than WASP. Thus, we propose this switch to N-WASP during HCMV infection of monocytes promotes migration in the absence of a chemotactic gradient into organ tissues (Chan et al., 2009b). Rapid activation of the EGFR signaling pathway following HCMV infection also turns out to be essential for viral entry into monocytes [Figure 2 (Chan et al., 2009b; Fulkerson et al., 2020)]. Thus, EGFR plays an essential role in the pathobiology of HCMV by mediating viral entry into monocytes and stimulating the cellular signaling activity that promotes hematogenous dissemination of the virus.

The HCMV gB engages EGFR and then rapidly and directly activates downstream signaling via its intrinsic tyrosine kinase (Walker et al., 1998). gB mainly interacts with EGFR on monocytes, although in some cell types, it may also interact with cellular integrins (Yurochko et al., 1992; Bentz and Yurochko, 2008; Nogalski et al., 2011, 2013; Collins-McMillen et al., 2016; Kim et al., 2016). This receptor-ligand engagement stimulates the activation of transcription factors, NF-κB (discussed in more detail below) and Sp-1 (Yurochko et al., 1995, 1997a,b) and numerous signaling pathways (Chan et al., 2012a; Campadelli-Fiume et al., 2016; Eberhardt et al., 2016; Collins-McMillen et al., 2017b; Yurochko, 2017). In addition to activating signaling molecules and transcription factors, these functional changes initiated by HCMV gB binding to EGFR serve as a molecular convergence point linking the changes in molecular and biochemical pathways with the biological changes required for productive infection. One example is the viral activation of STAT1 (Collins-McMillen et al., 2017a, b). Not only is STAT-1 activated within minutes of viral binding, as well as chronically (3 plus weeks after infection) as documented by changes in the patterns of appropriate activating phosphorylated residues, its specific activation is required for the survival and differentiation of monocytes that ultimately is needed for productive infection and viral persistence in organ macrophages (Collins-McMillen et al., 2017b).

The HCMV infection elicits a robust immune response (Gerna et al., 2005; Sinzger et al., 2006; Mason et al., 2012). However, symptomatic infections largely only occur in immunocompromised patients (Stagno et al., 1986; Bentz et al., 2006; Arvin, 2007; Nogalski et al., 2014; Fulkerson et al., 2021). Thus, the immune response keeps the virus under tight control in healthy individuals. How viral infection and the immune response interacts during life-long persistence remains unresolved? With monocytes playing a central role during infection, understanding how the virus manipulates EGFR signaling pathways in monocytes (Yurochko, 2008; Stevenson et al., 2014), promotes reactivation (Streblow and Nelson, 2003; Nogalski et al., 2014; Collins-McMillen et al., 2018a, 2019), enhances production of suppressive cytokines (Chan et al., 2009a; Wu et al., 2017; Mikell et al., 2019) and alters differentiation and phenotypic polarization of monocytes and macrophages (Chan et al., 2008a, 2012a; Collins-McMillen et al., 2017a, b) could pave the way for a better understanding of the pathobiology of the virus.

#### Integrins

We previously described that viral engagement of β1 and β3 integrins are essential for infection of human peripheral monocytes via the interaction of the HCMV gH/gL/UL128-131 complex with these two integrin families [Figure 2 (Nogalski et al., 2013; Kim et al., 2016)]. It is also known from other groups that HCMV binds to integrins and facilitates viral entry into human fibroblasts (Compton, 2000; Wang et al., 2005; Campadelli-Fiume et al., 2016). However, we have noted that there are functional changes that occur during monocytes infection, including the requirement for multiple integrins, that was not observed in fibroblasts (Nogalski et al., 2013; Campadelli-Fiume et al., 2016). That is, engagement of both β1 and β3 integrins are required during viral attachment to mediate entry and outside-in signaling in monocytes (Campadelli-Fiume et al., 2016). HCMV binding to integrins on monocytes rapidly activates the integrin/c-Src signaling pathway to induce PI(3)K and other essential signaling pathways.

The HCMV binding to host cell receptors induces signals in a distinct manner for each cell type. In monocytes, this receptor-ligand engagement results in cellular differentiation and long-term cellular survival (Smith et al., 2004b; Chan et al., 2008a; Yurochko, 2008; Campadelli-Fiume et al., 2016); but how are integrins involved? Integrins are a family of heterodimeric receptors composed of a single α-chain and a single β-chain that when engaged via their various cognate ligands leads to the induction of specific signaling pathways via activation of c-Src and other tyrosine kinases (Yamada and Miyamoto, 1995; Hynes, 2002). Based on transcriptome analysis, integrin-mediated signaling plays a central role as a convergent point linking the specific cellular molecules that have been activated by HCMV viral binding on monocytes. For example, the integrin-mediated rapid phosphorylation of paxillin identified that paxillin is both required for HCMV entry and for the hematogenous dissemination of the virus in monocytes through altered motility (Nogalski et al., 2011). Paxillin is an adaptor protein and an important signal transducer in the regulation of actin rearrangement during cellular adhesion and movement. HCMV-infected human primary monocytes showed heightened levels of paxillin mRNA and protein expression in an integrin-dependent manner (Nogalski et al., 2011). The use of siRNA knockdown in monocytes revealed that paxillin is essential for virus-induced motility and viral entry (Nogalski et al., 2011). Thus, the activation of the integrin/c-Src/paxillin signaling pathway is essential for triggering the cellular changes that allow for HCMV entry and hematogenous dissemination.

It is important to note that both the gB/EGFR and the pentamer/integrin signaling axes drive independent events, as well as cooperate to modify the infected cell. For example, as discussed briefly in this review, integrins activate paxillin through integrin/c-Src activation (Nogalski et al., 2011), and EGFR separately regulates the expression of the N-WASP (Chan et al., 2009b). Together, both are required for activation of motility, since inhibition of either pathway block infected monocyte motility and migration. From this data, HCMV-cell interactions emerge as an essential trigger for the cellular alterations that allow for HCMV entry and hematogenous dissemination.

### Extended Cellular Signaling and Intracellular Trafficking During HCMV Infection of Monocytes

An investigation into receptor-ligand mediated signaling and the associated regulation of survival and differentiation led us to a finding that cellular signaling during viral binding also controls unique post entry events; these include the intracellular trafficking and nuclear translocation of the virus during infection of monocytes (Figure 2). All herpesviruses replicate their DNA in the nucleus, and thus the steps of intracellular trafficking and nuclear translocation are essential steps following viral entry for productive infection. As this brief discussion will show, the virus has evolved to utilize the same signaling pathways required for entry to modulate, in a cell specific manner, these key post entry events. Briefly, we observed in monocytes that the gH/gL/UL128-131 complex through binding to β1 and β3 integrins and activation of c-Src signaling drives a pattern of intracellular trafficking and nuclear translocation not seen in other cell types. That is, HCMV nuclear translocation in monocytes is distinct from that seen in fibroblasts, endothelial cells, and CD34^+^ cells (Kim et al., 2016; Fulkerson et al., 2020). Nuclear translocation occurs roughly 30 min post infection in fibroblasts and endothelial cells (Kim et al., 2016). In contrast, post entry of viral DNA into the nucleus of monocytes is extended until 3 days post infection (dpi) (Kim et al., 2016; Fulkerson et al., 2020). Furthermore, we showed that mature HCMV particles are initially retained in early endosomes, they then move to the *trans*-Golgi network (TGN) and then finally to recycling endosomes in monocytes, where de-envelopment occurs (Kim et al., 2016). We recently added to this work and showed that gB/EGFR binding continues past the entry process and that this chronic gB/EGFR engagement was essential for nuclear translocation following the integrin/pentamer/c-Src decision point (Fulkerson et al., 2020). That is, viral engagement of EGFR continues from the attachment step through the post entry phase in order to promote correct viral tracking and nuclear translocation in monocytes (Fulkerson et al., 2020). Although studied in less detail, it seems to be a similar, although expedited, process occurs in CD34^+^ HPCs (Kim et al., 2017). This unique signaling pathway suggests that HCMV-infected monocytes use a distinctive mechanism in infected monocytes to promote productive infection and that this process is controlled by pentamer/integrin and gB/EGFR engagement.

## Monocyte-To-Macrophage Differentiation Requires Regulation of Monocyte and Macrophage Apoptotic Programs During HCMV Infection

**FIGURE 3:**
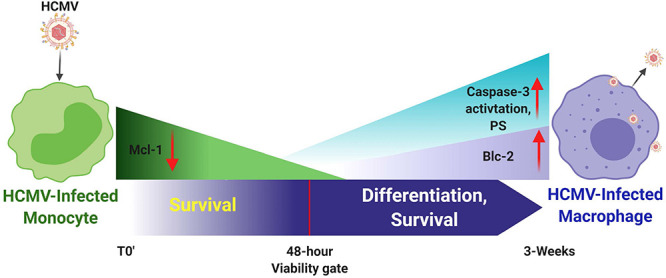
Human cytomegalovirus infection of monocytes drives a number of key molecular mechanisms required to enhance survival of normally short-lived monocytes and for the promotion of the differentiation of these infected monocytes into long-lived productively infected macrophaqes. It is important to note that monocytes, although they can be infected, they are not permissive for viral replication; only upon differentiation is *de novo* viral gene expression observed. HCMV-infected monocytes show enhanced expression of Mcl-1 early after infection (<48 h post infection) to prolong survival through the key 48-h viability gate. Mcl-1 levels then gradually decrease through this 48 h viability gate. Enhanced expression of Bcl-2 is then observed, as well as partial activation of caspase-3, which together seem to promote both long term survival and differentiation of these infected monocytes toward viral replication-permissive tissue macrophages that begin to produce mature virus around 3 weeks after infection.

Monocyte-to-macrophage differentiation in HCMV-infected monocytes is essential for long-term viral persistence. Monocytes have a short lifespan of 1–3 days in circulation and are not initially permissive for viral gene expression and replication (Min et al., 2020). Thus, HCMV must extend the life span of the infected monocytes and promote differentiation of these cells into long lived macrophages that are permissive for viral replication (Min et al., 2020). Briefly, as described in the mentioned review and associated references (Min et al., 2020), HCMV binds to and enters monocytes and initiates the process of nuclear translocation; simultaneously to the viral manipulated events that are essential to getting the genome into the nucleus, the virus must promote survival and differentiation of that infected monocyte. Only in the differentiated cell (the macrophage) does viral gene expression initiate along with all of the other steps associated with productive infection. This process is essential for organ infection following primary infection (Min et al., 2020) and following reactivation of virus from latency. Figures 2, 3 show the signaling associated with these discussed biological changes. Mcl-1, a member of the Bcl-2 gene family, serves to regulate monocyte survival (Yang et al., 1996; Zhou et al., 1997). HCMV uses the EGFR/PI3K signaling pathway to upregulate this anti-apoptotic protein after infection; thus, overcoming the intrinsic apoptotic fate of monocytes (Chan et al., 2010). Mcl-1 was upregulated/maintained early in HCMV-infected monocytes (Chan et al., 2010) and then begins to diminish to near background levels around 48 h post infection (hpi). Reeves et al. (2012) have also shown the induced expression of Mcl-1 during monocyte infection. siRNA-mediated knockdown of Mcl-1 revealed the strong anti-apoptotic role that Mcl-1 plays in HCMV-infected monocytes during the first 48 hpi (Chan et al., 2010). Additional studies showed that Bcl-2 then takes over for Mcl-1 in monocytes after the first 48 hpi (Chan et al., 2010; Stevenson et al., 2014; Collins-McMillen et al., 2016). Bcl-2 is known to be an anti-apoptotic factor in the survival of myeloid cells (Lin et al., 1996; Lagasse and Weissman, 1997; Sanz et al., 1997; Adams and Cory, 1998; Perlman et al., 2000; Liu et al., 2001; Zhang et al., 2003; Reeves et al., 2012; Zhu et al., 2018; Shnayder et al., 2020). Molecularly, known to be anti-apoptotic, their regulation and linear regulation (Mcl-1 and then Bcl-2) is not seen in mock infected cells and monocytes treated with other stimuli (Zhou et al., 1997; Collins-McMillen et al., 2016). Both of these factors are induced by cellular signaling with Mcl-1 early regulation being controlled by the gB/EGFR axis and the later Bcl-2 upregulation being largely controlled by the pentamer/integrin axis (Zhou et al., 1997; Chan et al., 2010; Reeves et al., 2012; Collins-McMillen et al., 2016). To add to the potential complexities of the control of monocyte survival, HCMV seems to block full caspase-3 activation, while inducing partial activation of caspase-3, which in turn is important for monocyte-to-macrophage differentiation (Chan et al., 2012b). Thus, it seems that HCMV modulation of monocyte survival is related to monocyte-to-macrophage differentiation (discussed below) and these combined major biological changes, usurped during infection of monocytes, are essential for productive organ infection and long-term persistence of the virus in a variety of organ macrophages (Min et al., 2020).

## Unique Cellular Signaling Pathways Induce Monocyte-To-Macrophage Differentiation Likely Required for Productive Infection in Peripheral Organs

Because infected monocyte-to-macrophage differentiation is required for viral replication and long-term viral persistence, we have focused on the control of differentiation of monocytes into macrophages and how cellular signaling via gB-EGFR engagement controls this process. In general, macrophage phenotypes have been classified as M1 and M2 (Chan et al., 2009a; Italiani and Boraschi, 2014). M2 macrophages have been further classified into M2a, M2b, and M2c (Mantovani et al., 2004). M1 macrophages are usually considered to be classically activated cells and produce proinflammatory cytokines and these cells help initiate immune responses (Mantovani et al., 2004; Sica and Mantovani, 2012). M2 macrophages are usually considered to be alternatively activated cells and generally produce anti-inflammatory cytokines or participate in events such as wound healing (Mantovani et al., 2004; Lawrence and Natoli, 2011; Murray and Wynn, 2011; Tugal et al., 2013). HCMV infection of monocytes stimulates the differentiation of infected cells toward macrophages (Figure 3) that are polarized toward an M1 proinflammatory phenotype that we argue is a critical step for viral persistence (Smith et al., 2004a; Min et al., 2020). It is important to note that these infected macrophages, although biased toward M1 (based on ‘omics profiling), also show aspects of M2 polarization, especially the production of some anti-inflammatory and immune inhibitory cytokines (Smith et al., 2004a; Chan et al., 2008a; Min et al., 2020). For example, analysis of chemokine production by transcriptome and secretome analysis revealed that HCMV-infected monocytes exhibit a distinct reprogramming of their differentiation and polarization with more M1-associated gene transcripts being upregulated than M2-associated gene transcripts. Similarly, M1-associated chemokines were also more dominantly secreted compared to M2-associated chemokines in HCMV-infected monocytes (Chan et al., 2008a, 2009a). In both of these ‘omics analyses, we saw a bias toward an M1 phenotype, but not an absolute, as the infected cells appeared to be unique M1/M2 polarized macrophages expressing key elements of both polarization potentials. It seems that the virus favors the aspects of both M1 and M2 that promote persistence (Stevenson et al., 2014). That is, the proinflammatory aspects are useful to allow monocytes to move from blood to tissue and differentiate in macrophages that can be productively infected and that can recruit additional monocytes while retaining the ability to secrete immune inhibitory products and prevent T and B cell infiltration (Chan et al., 2008a, 2009a). Flow cytometry analysis has also shown that both M1- and M2-associated macrophage markers were increased in HCMV-infected monocytes when compared to mock-infected control monocytes (Chan et al., 2008a, 2009a; Stevenson et al., 2014; Collins-McMillen et al., 2017a, b). Viral receptor-ligand events dictate these changes - that is the gB/EGFR and pentamer/integrin signaling are key to dictating and initiating this process of differentiation and polarization (Chan et al., 2012a, b; Min et al., 2020).

The above-mentioned changes occur during primary or direct infection of freshly isolated blood monocytes. Because monocytes are also an essential player in the process of reactivation from latency, where infected CD34^+^ HPCs differentiate during reactivation into monocytes, there is also an intense interest in understanding the phenotypic and molecular underpinnings of these cells during reactivation. Differences appear to exist in the nature of these monocytes during reactivation and this is likely due to the variable nature of these cells. It has been argued that reactivating monocytes express a proinflammatory biology (Goodrum et al., 2002; Streblow and Nelson, 2003; Sinclair, 2008) and/or an immunosuppressive or anergic state as described by Zhu et al. (2018) and Shnayder et al. (2020). Together, this combined biological data suggest that HCMV directs the nature of the differentiation and the M1- and M2-polarized phenotypes (Stevenson et al., 2014), and that this end polarization phenotype does not fall into a strict M1- or M2-like phenotype or perhaps does not fall into a strict proinflammatory or immunosuppressive state.

## HCMV Modulates NF-κB Cellular Signaling

NF-κB is essential for initial HCMV gene expression since the HCMV major immediate early promoter requires NF-κB (Fields and Knipe, 2013; Fields et al., 2013). This early identification of the impact of NF-κB on the initiation of HCMV gene expression served as the impetus to drive early studies into the regulation of NF-κB. It has been shown that HCMV activates both the canonical and non-canonical NF-κB signaling pathways (Figure 2) to aid in viral gene expression, viral replication, and productive infection (Andreoni et al., 2002; Chan et al., 2008b, 2012a; Yurochko, 2008; Collins-McMillen et al., 2017b). From a regulatory standpoint, it was shown in both fibroblasts and monocytes that gB- and gH-mediated signaling induces rapid activation of NF-κB signaling pathways (and degradation of IκBα); only in monocytes, however, did this rapid activation of NF-κB lead to increased inflammatory cytokine production, motility, endothelial cell adhesion, and transendothelial migration (Yurochko et al., 1997a, 1999; Smith et al., 2004b, 2007; Yurochko, 2008; Chan et al., 2009b), suggesting cell type-specific effects. Because viral-induced changes in NF-κB seem to control cellular function in monocytes separate from the direct role on viral gene expression, NF-κB activation and its role in changes in infected monocyte function remain a topic of interest. Furthermore, because NF-κB controls multiple aspects of hematopoiesis and cellular development (Hancock et al., 2017; Zhang et al., 2017), it is likely the receptor-ligand changes in this transcription factor may help explain some of the broad biological changes described in the sections above. ‘Omics profiling is consistent with this idea, where inhibition of NF-κB signaling blocked or down-regulated over 500 genes at 4 hpi, including multiple gene products associated with apoptosis, motility, and differentiation (Chan et al., 2008b). These observations imply that modulation of NF-κB signaling serves as a cell-specific convergence point where viral activation mediated by receptor-ligand engagement through EGFR and integrins stimulates a cell in a specific manner to promote the required changes for infection of that cell. It is important to point out that the viral miRNAs miR-US5-1 and miR-UL112-3p target the IκB kinases IKKa and IKKb for downregulation and can negatively influence proinflammatory cytokine expression suggesting additional mechanisms by which HCMV may specifically control this key pathway or specific parts of this pathway during lytic and/or latent infection (Hancock et al., 2017). Other transcription factors have also been reported to be regulated by receptor-ligand signaling and during key points of latent or lytic infection cycle (Fields and Knipe, 2013; Fields et al., 2013). The entirety of this process is beyond the scope of the current review.

## Host Cell Signaling and Latency

**FIGURE 4:**
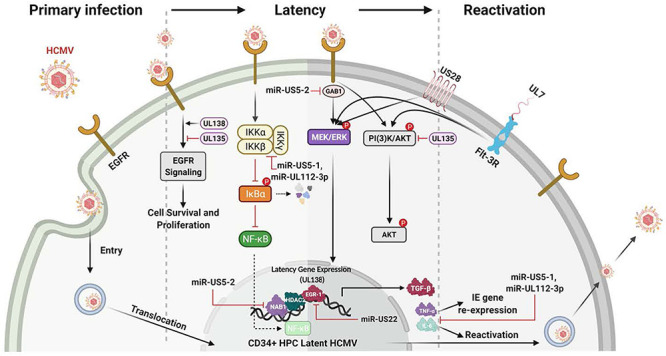
Many HCMV-expressed proteins and miRNAs regulate the signaling pathways known to be important for the establishment, maintanence and reactivation from latency. The initial infection of CD34^+^ HPCs lays the foundation for the establishment of latency. Initially, gB engagement of EGFR and the ensuing signaling is required for HCMV entry into CD34^+^ HPCs. This initial ligand-dependent event prepares the cell for the establishment of latency through the manipulation of host signaling pathways and regulation of viral latent gene expression. These latency gene products in turn are critical for the establishment, the maintenance of and/or reactivation from latency. For example, gene products such as UL138 and UL135 regulate viral latency and host interactions by regulating various signaling pathways [such as Egr-1 activity or PI(3)K signaling] during the establishment of and exit from latency. Other products such as the viral miRNAs miR-US5-1 and miR-UL112-3p target and down regulate IKKα and IKKβ to control inflammatory cytokine regulation (such as IL-6 and TNFα) and likely to mitigate NF-KB dependent signaling that might interfere with latency programming. Other products such as soluble UL7 are released during reactivation and through binding to the Flt-3R induces activation of the downstream PI3K/AKT and MAPK/ERK signaling pathways that in turn triggers the HPC and monocyte differentiation required for complete viral reactivation. US28, another critical latency modulator, is involved in the MEK/ERK and EGFR/PI3K signaling required for HCMV reactivation from latency, as well as playing a key role in monocyte to macrophage differentiation. In addition, a variety of viral miRNAs (such as miR-US5-1, miR-US5-2, miR-US22, and miR-UL112-3p) target cellular processes and regulate virus-mediated signaling. As examples, miR-US5-2 blocks NAB1 activity, a represser of TGFβ resulting in increased production of the TGFβ that suppresses hematopoietic events; miR-US5-2 also down regulates GAB1, thus lowering MEK/ERK signaling, as well as regulates EGR1 and UL138 expression supporting a role for miR US5-2 during reactivation. Overall these viral miRNAs, UL7, US28, UL138/135 control vital signaling pathways during the establishment, maintenance of and during reactivation from latency and are intimately involved in the differentiation of the infected CD34^+^ HPCs into monocytes and then into productive tissue macrophages.

The HCMV latency and reactivation are crucial for long-term persistence and stable spread within the human population (Jarvis and Nelson, 2002; Streblow and Nelson, 2003; Petrucelli et al., 2009; Buehler et al., 2016; Collins-McMillen and Goodrum, 2017; Kim et al., 2017; Collins-McMillen et al., 2018a, 2019; Hancock et al., 2020a). HCMV latency also exploits cellular signaling pathways to help establish and maintain latency and then upon recognition of appropriate signals, to manipulate the cellular environment and allow successful viral reactivation and differentiation of the infected CD34^+^ HPCs to blood monocytes and then to tissue macrophages (Jarvis and Nelson, 2002; Petrucelli et al., 2009; Collins-McMillen et al., 2018a, 2019; Hancock et al., 2020a). As with primary infection, EGFR signaling is also an important cellular receptor controlling this aspect of the viral infection process (Figure 4). HCMV activation of EGFR and downstream PI3K signaling is important for viral entry into CD34^+^ HPCs (Kim et al., 2017). Furthermore, through the use of the EGFR inhibitor, AG1478, it was shown that inhibition of EGFR signaling post entry resulted in increased transcription of IE1/IE2, while curbing the transcription of the latency-associated UL138 transcript (Kim et al., 2017). EGFR is also essential for the maintenance of latency, with the virus controlling a low level of constant signaling to maintain stable latency (Buehler et al., 2019; Mikell et al., 2019); this will be discussed in more detail below. Thus, viral signaling during attachment and entry is important in a distinct manner in numerous cell types essential for viral persistence; while long-term regulation of EGFR signaling and possibly integrin signaling post entry is critical for latency and persistence. Cellular differentiation (Min et al., 2020) is also closely related to both latency and reactivation and one expects multiple aspects of altered signaling during reactivation and latency to be central to the process in much the same way that signaling is essential for the establishment of persistence during primary infection.

### The EGFR and pUL135 and pUL138 Connection

UL135 and UL138 are two key viral genes in the ULb’ region important in latency and reactivation. They have been shown to have opposing roles in regulating EGFR’s function in viral replication/reactivation (Umashankar et al., 2014; Buehler et al., 2016). The ULb’ region encodes the UL133-UL138 locus, which is required for infection and persistence in the host. The UL133, UL135, UL136, and UL138 genes have been shown to have important functions in vascular endothelial cells (Bughio et al., 2013, 2015) and to differentially regulate latency and reactivation in CD34^+^ HPCs (Petrucelli et al., 2009, 2012; Umashankar et al., 2011, 2014). pUL135 appears to promote replication, via inhibition of EGFR signaling through the turnover of EGFR from the cell surface. UL135 promotes endocytic trafficking of EGFR for lysosomal degradation through its interaction with the adapter proteins, Abelson interactor 1 (ABI-1) and SH3 domain-containing kinase-binding protein 1 (CIN85) (Rak et al., 2018). Loss of UL135 or disruption of its interaction with Abi-1 or CIN85 results in increased cellular and surface levels of EGFR and a failure to reactivate from latency (Buehler et al., 2016; Rak et al., 2018). In contrast, pUL138 activity is required for continued surface expression of EGFR (Buehler et al., 2016). Thus, in this model, pUL135 and pUL138, along with EGFR signaling, comprise a molecular convergence point to control latency and reactivation.

### Viral MicroRNAs (miRNAs) and Host Signaling

Viruses encode miRNAs that regulate viral and host gene expression to generate a more favorable cellular environment and/or inhibit host immune responses. Several herpesvirus family members express miRNAs that regulate host cell signaling pathways (Gottwein and Cullen, 2008; Hook et al., 2014; Hancock et al., 2020a). The 14 HCMV miRNAs (Stark et al., 2012) are unique among human herpesviruses because they are not clustered within defined latency-associated genomic regions and thus are differentially expressed during lytic and latent infection (Dunn et al., 2005; Pfeffer et al., 2005; Buck et al., 2007; Dolken et al., 2007). Overall, herpesvirus miRNAs target broadly similar cellular functions, including secretory pathways, immune evasion, survival, and proliferation of infected cells. A number of the HCMV miRNAs control the signaling pathways associated with long-term viral persistence; some of these key miRNAs are briefly discussed below.

HCMV miR-US5-1, miR-US5-2, and miR-UL112-3p down-modulate secretion of the inflammatory cytokines tumor necrosis factor-alpha (TNF-α) and interleukin-6 (IL-6), by targeting multiple members of the endocytic secretory pathway (Hook et al., 2014). In addition, miR-US5-1 and miR-UL112-3p block NF-κB-dependent induction of proinflammatory cytokines through direct targeting of the IκB kinase (IKK) complex components IKKα and IKKβ (Hancock et al., 2017). It has also been determined that miR-UL112-3p can modulate the Toll-Like Receptor (TLR) innate immune pathway through direct targeting of TLR2. Because TLRs play a critical role in controlling viral infections (Akira et al., 2006), this altered signaling could also be involved in viral persistence. Specifically, it was shown that miR-UL112-3p efficiently down-regulated the TLR2/IRAK1/NF-κB signaling axis (Landais et al., 2015).

The HCMV reactivation from latency can cause significant disease in transplant recipients (Safdar et al., 2002; Ljungman et al., 2011; Hancock et al., 2020a). Clinical manifestations include myelosuppression and graft rejection as a result of infection of CD34^+^ HPCs (Fries et al., 1993). Mechanistically, little had been uncovered to account for the global suppression caused by infection of only a small number of CD34^+^ HPCs, until a recent study examining the regulation of TGF-β. TGF-β is a member of a multifunctional cytokine family responsible for many cellular processes (Marshall et al., 2000; Capron et al., 2010; Blank and Karlsson, 2015), including potent inhibition of early progenitor cell proliferation (Sing et al., 1988). Recently it was shown that latently expressed HCMV miR-US5-2 downregulates NAB1, a repressor of the transcriptional factor NGFI-A (Egr-1), which in turn results in increased TGF-β production that causes myelosuppression in CD34^+^ HPCs. These results implicate viral miRNAs in regulating HCMV-induced myelosuppression during solid organ and hematopoietic stem cell transplantation (Hancock et al., 2020a). Viral miRNAs can also influence viral gene expression. For example, miR-US5-1 and miR-US5-2 can both regulate HCMV US7 expression (Tirabassi et al., 2011) while miR-UL112-3p targets the immediate early protein IE1 (IE72) (Grey et al., 2007; Murphy et al., 2008), which has implications in latency establishment and immune cell recognition (Lau et al., 2016). Together, these examples show how the viral miRNAs can influence cell signaling and cytokine secretion that in turn promotes key elements of viral persistence.

So far, several studies have addressed how HCMV miRNAs influence EGFR and EGFR-dependent downstream signaling. HCMV miR-US5-2 targeting of the EGFR adaptor protein GAB1 results in a block in MEK/ERK signaling downstream of EGFR activation that affects both cell proliferation and expression of pUL138, thus interfering with the feed-forward loop of EGFR activation of pUL138 expression (Hancock et al., 2020b). pUL138 expression is also regulated by the HCMV miRNA miR-US22, which directly targets the transcription factor Egr-1 downstream of EGF-mediated MEK/ERK signaling. Through targeting Egr-1, miR-US22 regulates pUL138 expression and CD34^+^ HPC proliferation and plays an important role in viral reactivation (Mikell et al., 2019). With the role chronic EGFR signaling plays in the maintenance of latency (Buehler et al., 2016), it seems probable that these miRNAs will strongly tune the EGFR response by modulating signaling pathways to promote latency or, when appropriate, viral reactivation.

### UL7 as an HCMV Signaling Molecule

UL7 is a secreted HCMV-encoded signaling molecule known to play a robust role during lytic and latent infection (Engel et al., 2011; Crawford et al., 2018; Perez-Carmona et al., 2018; Min et al., 2020). UL7 belongs to the HCMV RL11 gene family (RL11-13, UL1, UL4-11, RL6, and RL5A) and signals through Fms like tyrosine kinase 3 receptor (Flt-3R), a Class III receptor tyrosine kinase predominantly expressed in HCMV infection of myeloid lineage cells, such as CD34^+^ HPCs or monocytes (Beaudin et al., 2014).

Flk2/Flt3 promotes both myeloid and lymphoid development by expanding non-self-renewing multipotent hematopoietic progenitor cells (Beaudin et al., 2014). The binding of HCMV UL7 to the Flt-3R triggers HPC/monocyte differentiation via PI3K and MAPK/ERK1/2 signaling (MacManiman et al., 2014; Crawford et al., 2018). UL7 has also been shown to promote angiogenesis (MacManiman et al., 2014) and alter cytokine production (Engel et al., 2011). The differences in UL7’s effect on monocytes vs. other cell types suggest its effects on signaling may vary from cell type to cell type. Deletion of UL7 from the virus results in loss of reactivation from latency *in vitro* and in humanized mice, further highlighting the functional role of UL7 during viral reactivation (Crawford et al., 2018; Hancock et al., 2020a). Finally, it was recently published that HCMV utilizes secreted protein pUL7, miR-US5-1, and miR-UL112-3p to reduce the proapoptotic transcription factor FOXO3a, which in turn reduces expression of proapoptotic gene BCL2L11 and prevents virus-induced apoptosis after infection of CD34^+^ HPCs (Hancock et al., 2021).

### US28 HCMV Signaling Molecules

US28, one of the 4 HCMV encoded G-protein coupled receptors (GPCRs), has been documented to signal through a number of pathways and this chemokine receptor has a number of biological functions (Streblow et al., 1999; Miller et al., 2003, 2012; Vischer et al., 2006; Elder and Sinclair, 2019; Elder et al., 2019). US28 binds to both members of the CC and CX_3_C chemokine families making it unique among GPCRs; and furthermore, the receptor elicits ligand-specific signaling and functions that are also cell type specific (Vomaske et al., 2009). US28 can also signal in a ligand-dependent and -independent manner (Streblow et al., 2003; Miller et al., 2012). US28 has been shown to alter smooth muscle cell migration (Streblow et al., 1999) and to be important in vascular disease, as well as play a role during latency where US28 is argued to be key in both maintenance of and reactivation from latency (Crawford et al., 2019). US28 is expressed in naturally infected human peripheral blood cells during periods of latency (Krishna et al., 2017), reactivation in lung transplant recipients (Boomker et al., 2006), and in the model of HCMV latency using CD34^+^ HPCs, monocytes, and in monocyte-derived macrophages during active infection (Zipeto et al., 1999; Beisser et al., 2001; Goodrum et al., 2002; Cheung et al., 2006; Humby and O’Connor, 2015; Crawford et al., 2019). Overall, US28 regulates host cell signaling, which in many cases is through changes in PI3K/Akt and MAPK signaling and alterations in EGFR and downstream processes. Together these varied mechanisms briefly discussed along with others not discussed (Fields et al., 1996, 2013; Fields and Knipe, 2013) demonstrate the complex mechanisms controlling HCMV reactivation from latency and the intimate linking of these various viral gene products in controlling the cellular signaling required for reactivation.

## Conclusion

The HCMV has evolved a complicated strategy to disrupt biological pathways in infected host cells. The understanding of the relationship between viral infection and cellular signaling helps provide clues for how viral proteins and other regulators (whether they control primary infection or latency) hijack host cellular signaling pathways and control the pathways to the virus’ advantage. Deciphering the impact of cellular receptor engagement during viral entry and the corresponding exploitation of downstream host cellular signaling is vital to understanding viral dissemination and life-long viral persistence in individual hosts and in the human population in general. HCMV gB and gH complexes are critical for initiating these events through their binding to cognate host cell receptors during primary infection, while a number of viral proteins and miRNAs are critical to controlling the signaling associated with maintenance and reactivation from latency. Because each signaling event in each cell type is likely different, it is important to study the multiple cell types and how each event dictates the unique biology and infection characteristics within each cell. We argue this last point is evident during infection of monocytes and CD34^+^ HPCs, two critical cell types required for persistence and dissemination, where data emphasizes that what is seen in other cell types such as fibroblasts and epithelial and endothelial cells is not seen during infection of these two cell types and vice versa. Overall, we believe that by defining the HCMV signaling pathway activated during infection, it will be possible to develop new treatment options for viral diseases.

## Author Contributions

All authors listed have made a substantial, direct and intellectual contribution to the work, and approved it for publication.

## Conflict of Interest

The authors declare that the research was conducted in the absence of any commercial or financial relationships that could be construed as a potential conflict of interest.
